# A Hybrid Deep Learning–Based Feature Selection Approach for Supporting Early Detection of Long-Term Behavioral Outcomes in Survivors of Cancer: Cross-Sectional Study

**DOI:** 10.2196/65001

**Published:** 2025-03-13

**Authors:** Tracy Huang, Chun-Kit Ngan, Yin Ting Cheung, Madelyn Marcotte, Benjamin Cabrera

**Affiliations:** 1 Emory University Atlanta, GA United States; 2 Worcester Polytechnic Institute Worcester, MA United States; 3 Chinese University of Hong Kong Hong Kong China (Hong Kong); 4 Arizona State University Tempe, AZ United States

**Keywords:** machine learning, data driven, clinical domain–guided framework, survivors of cancer, cancer, oncology, behavioral outcome predictions, behavioral study, behavioral outcomes, feature selection, deep learning, neural network, hybrid, prediction, predictive modeling, patients with cancer, deep learning models, leukemia, computational study, computational biology

## Abstract

**Background:**

The number of survivors of cancer is growing, and they often experience negative long-term behavioral outcomes due to cancer treatments. There is a need for better computational methods to handle and predict these outcomes so that physicians and health care providers can implement preventive treatments.

**Objective:**

This study aimed to create a new feature selection algorithm to improve the performance of machine learning classifiers to predict negative long-term behavioral outcomes in survivors of cancer.

**Methods:**

We devised a hybrid deep learning–based feature selection approach to support early detection of negative long-term behavioral outcomes in survivors of cancer. Within a data-driven, clinical domain–guided framework to select the best set of features among cancer treatments, chronic health conditions, and socioenvironmental factors, we developed a 2-stage feature selection algorithm, that is, a multimetric, majority-voting filter and a deep dropout neural network, to dynamically and automatically select the best set of features for each behavioral outcome. We also conducted an experimental case study on existing study data with 102 survivors of acute lymphoblastic leukemia (aged 15-39 years at evaluation and >5 years postcancer diagnosis) who were treated in a public hospital in Hong Kong. Finally, we designed and implemented radial charts to illustrate the significance of the selected features on each behavioral outcome to support clinical professionals’ future treatment and diagnoses.

**Results:**

In this pilot study, we demonstrated that our approach outperforms the traditional statistical and computation methods, including linear and nonlinear feature selectors, for the addressed top-priority behavioral outcomes. Our approach holistically has higher *F*_1_, precision, and recall scores compared to existing feature selection methods. The models in this study select several significant clinical and socioenvironmental variables as risk factors associated with the development of behavioral problems in young survivors of acute lymphoblastic leukemia.

**Conclusions:**

Our novel feature selection algorithm has the potential to improve machine learning classifiers’ capability to predict adverse long-term behavioral outcomes in survivors of cancer.

## Introduction

### Background

The number of survivors of cancer is increasing globally. The American Cancer Society recently reported that in 2023, a total of 1,958,310 new cancer cases were projected to occur in the United States [1]. Treatment advances have resulted in a dramatic improvement in the survival rates of most cancers, especially in resource-limited countries and regions. However, this growing population of survivors of cancer may develop a myriad of treatment-related adverse effects that lead to a compromised health status. Studies have also shown that survivors of cancer are more likely than the general population to experience negative long-term behavioral outcomes, such as anxiety, depression, attention problems, and sluggish cognitive tempo, after cancer treatments [2]. Contemporary treatment strategies have led to improved life expectancy after treatment for pediatric cancer, especially in survivors of acute lymphocytic leukemia (ALL) [3]. Given that studies have shown that the promotion of a healthy lifestyle and interventions that reduce physical and mental health burdens can lead to reduction in all-cause and cause-specific mortality, addressing the risk factors of adverse functional outcomes early on is critical [4-6]. Thus, developing an effective approach to identify crucial factors and then detect these negative outcomes in advance is needed so that medical therapists can intervene early and take the appropriate actions and treatments promptly to mitigate adverse effects in survivors of cancer.

### Current Approaches for Detecting Adverse Behavioral Outcomes in Survivors of Cancer

Currently, to support the identification of relevant factors and the early detection of adverse behavioral outcomes for survivors of cancer, clinical scientists use various statistical analyses to understand the relationship among those behavioral outcomes, cancer treatments, chronic health conditions, and socioenvironmental factors [7-9]. Specifically, traditional statistical methods (ie, linear regression analysis) are used to extract predictor variables and then model the relationship between the extracted predictor variables and the behavioral outcomes. This analysis assumes that the behavioral outcomes are, for the most part, linearly correlated with those predictor variables. However, this assumption may not always hold in this complex and dynamic problem. Furthermore, the predictors for those behavioral outcomes extracted by statistical methods may have weak prediction accuracy, as modeling human behavioral outcomes is challenging due to its multifactorial nature (ie, many predictors as well as interactions among the predictors affecting the outcome), heterogeneity (ie, differences across individuals), nonlinearity of data, multicollinearity (ie, highly correlated variables), class imbalance (ie, few observations of the outcome of interest), and missing data [10,11]. As a result, this class of linear regressors can only account for a small proportion of variance, with limited usability in a clinical setting. Thus, developing an effective computational methodology that can maximize the use of those data for prognostic and predictive behavioral outcomes is highly desirable.

To address the abovementioned problems, feature selection techniques in machine learning (ML) play an important role. Feature selection techniques can be broadly divided into 4 categories: filter, wrapper, embedded, and hybrid. Filter methods select features based on their statistical significance to the outcome of interest. Unlike other feature selection methods, such as wrapper and embedded methods, filter methods function independent of any ML classifiers. However, filter methods are less accurate than other methods of feature selection, such as wrapper methods. In addition, there is a risk of selecting redundant features when using filter methods that do not consider the correlation between features. Wrapper methods use a greedy search algorithm (ie, an iterative algorithm that makes the locally optimal choice at each step) with a classifier to sequentially add and remove features from the classifier to maximize the specified scoring metrics, that is, precision, recall, and *F*_1_-score. The output is the best subset of features that the algorithm found. While wrapper methods are proficient in achieving high classification accuracy, they are not efficient in computation time or complexity. In addition, there is also a risk of overfitting with wrapper methods, where the classifier is highly trained to generate accurate predictions for the training data only and cannot correctly create generalized predictions for testing data or any novel datasets. Embedded methods use qualities from both filter and wrapper methods to perform feature selection during the construction of the ML classifiers. The baseline embedded methods that are commonly used are least absolute shrinkage and selection operator (Lasso), Ridge, and ElasticNet. However, to effectively use embedded methods, prior knowledge of the feature sets is required. In addition, embedded methods could pose problems when identifying small feature sets. Hybrid methods combine filter and wrapper methods to take advantage of the benefits each method provides, while minimizing their limitations [12]. A filter method first selects a subset of features, which are then input into a wrapper method to further select the best subset of features. As hybrid methods are a combination of filter and wrapper methods, they inherit problems from both—filter methods may exclude important features and wrapper methods are inefficient in computation time.

### Goal of This Study

To bridge the abovementioned gaps, we propose a hybrid deep learning–based feature selection approach to support early detection of long-term adverse behavioral outcomes in survivors of cancer. Specifically, our goals are four-fold: (1) devise a data-driven, clinical domain–guided framework to select the best set of features among cancer treatments, chronic health conditions, socioenvironmental factors, and others; (2) develop a 2-stage feature selection algorithm, that is, a multimetric, majority-voting filter and a deep dropout neural network (DDN), to dynamically and automatically select the best set of features for each behavioral outcome; (3) conduct an experimental case study on our existing study data with 102 survivors of ALL (aged 15-39 years at evaluation and >5 years postcancer diagnosis) who were treated in a public hospital in Hong Kong; and (4) design and implement radial charts to illustrate the significance of the selected features on each behavioral outcome to support clinical professionals’ future treatment and diagnoses. In this pilot study, we demonstrate that our approach outperforms the traditional statistical and computation methods, including linear and nonlinear feature selectors, for the addressed top-priority behavioral outcomes.

## Methods

### Review of Baseline Feature Selection Methods

#### Overview

Four baseline feature selection methods were used in the experimental studies as a comparison for our novel feature selection algorithm (Textbox 1).

**Summary of the baseline feature selection methods.**

**Filter**

- Correlation-based feature selection (CFS)
- Information gain (IG)
- Maximum relevance minimum redundancy (MRMR)

**Wrapper**

- Sequential forward selection (SFS)
- Sequential backwards selection (SBS)
- Stepwise selection (SS)

**Embedded**

- Least absolute shrinkage and selection operator (Lasso)
- Ridge
- ElasticNet

**Hybrid**

- CFS→SFS
- IG→SFS
- MRMR→SFS
- CFS→SFS
- IG→SFS
- MRMR→SFS
- CFS→SBS
- IG→SBS
- MRMR→SBS
- CFS→SS
- IG→SS
- MRMR→SS

#### Filter Methods

Filter methods select features based on their statistical significance to the outcome of interest, independent of any ML classifiers. To evaluate the performance of existing filter methods, we use information gain (IG), maximum relevance minimum redundancy (MRMR), and correlation-based feature selection (CFS) [13]. IG is calculated by comparing the entropy of the dataset before and after a transformation. When IG is used for feature selection, it is called mutual information and works by evaluating the IG of each variable in the context of the target. The MRMR algorithm selects the best *K* features at each iteration that have maximum relevance with respect to the target variable and minimum redundancy with respect to the other features. The CFS algorithm involves splitting the features into subsets based on whether their values are continuous or discrete and can be used to measure the correlation between features and the target outcomes. For continuous data, Pearson correlation can be used, and for discrete data, symmetrical uncertainty can be used. Symmetrical uncertainty is a measure of relevance between features and targets that uses mutual information [14]. When evaluating the performance of the existing filter methods, we selected the top 15 features that had the highest scores for each of the 3 approaches.

#### Wrapper Methods

For binary classification, wrapper methods use a greedy search algorithm with a classifier to sequentially add and remove features from the classifier to maximize the specified scoring metric, that is, precision, recall, and *F*_1_-score. The output is the best subset of features that the algorithm found. To evaluate existing wrapper methods’ performances, we selected 3 commonly implemented wrapper methods: sequential forward selection (SFS), sequential backward selection (SBS), and stepwise selection (SS). SFS starts with an empty subset of features and iteratively adds features if adding them improves the specified score, according to the ML classifier. The selection terminates when a feature subset of the desired size *k*, where *k* refers to the number of features expected by the domain experts, is reached. In contrast, SBS starts with a full subset of all the features and iteratively removes features if removing them increases the specified score, according to the classifier. The selection also terminates when a feature subset of the desired size *k* is reached. SS, also known as bidirectional selection, alternates between forward and backward selection to select the best subset of features. To implement the wrapper selection approaches, we used the support vector machine classifier and used accuracy as the default scoring metric [15]. We also specified that the selection process should terminate when a feature subset of size 15 is reached. For the purpose of the study, we decided a priori that the feature subset should be limited to 15 because if there are too many exploratory factors in the model, the contribution of each factor to the variance may be too small and its clinical significance may be questionable.

#### Embedded Methods

Embedded methods use qualities from both filter and wrapper methods to perform feature selection during the construction of the ML classifier. The embedded classifiers we used were Lasso, Ridge, and ElasticNet. Lasso regression is a form of linear regression that imposes an L1 regularization penalty to identify the features that minimize the prediction error [16]. Similar to Lasso, Ridge regression is another form of linear regression that uses an L2 penalty instead [17]. ElasticNet regression merges Lasso and Ridge regression using the L1 and L2 regularization penalties [18]. ElasticNet regression can shrink some features to zero, similar to Lasso, while reducing the magnitude of other features, like Ridge. For each evaluated embedded method, we selected the top 15 most relevant features for each behavioral outcome.

#### Hybrid Methods

Hybrid methods combine filter and wrapper methods to take advantage of the benefits each method provides, while minimizing their limitations [12]. We implemented 9 different hybrid methods using the top 30 features selected from the 3 filter methods (ie, CFS, IG, and MRMR) and inputting them each into the 3 wrapper methods, including SFS, SBS, and SS, to subsequently select the top 15 features.

### Data-Driven, Clinical Domain–Guided Framework

**Figure 1:**
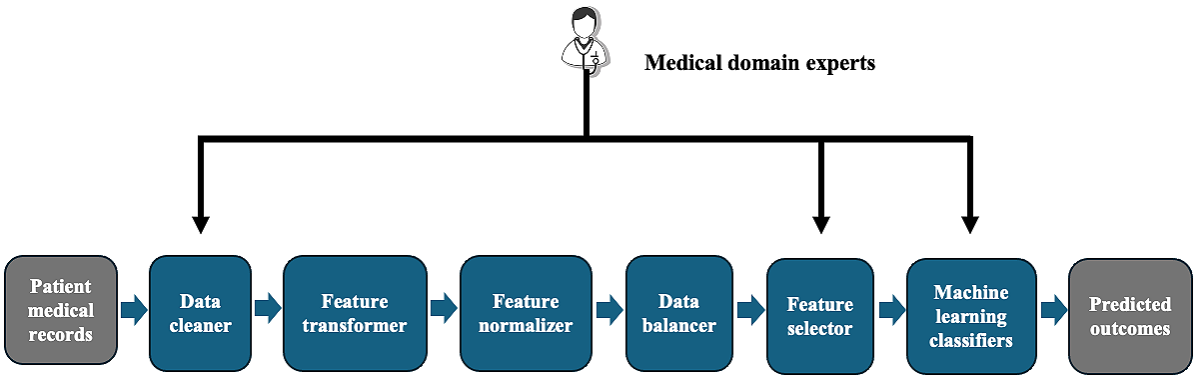
Data-driven, clinical domain–guided framework.

In this section, we describe and explain our framework that consisted of 6 main modules (Figure 1). The cancer survivor medical records, including the features, such as biomarkers, chronic health conditions, and socioeconomic factors, were first passed into the data cleaner that “sanitizes” the records with the clinical domain knowledge from our investigators. Note that throughout the framework, our clinical domain experts assisted us with certain processes. In this case study, for example, it consisted of replacing missing values in a patient’s record by averaging the existing values of the corresponding feature among all the other patients’ records grouped by a specific cancer type, age range, and biological sex. Clinical domain experts also helped us interpret and explain what different variable values mean for us to properly transform them into the correct variables.

Afterward, the records were passed into the feature transformer, where the one-hot encoding technique was used to transform categorical variables into binary ones [19]. For instance, we transformed the “gender” variable from categorical to binary by replacing “M” and “F” with 1 and 0.

Following feature transformation, the records were normalized by the feature normalizer. The Shapiro-Wilk test, the Kolmogorov-Smirnov test, and the D’Agostino-Pearson test were used to check whether features follow a normal distribution. If 2 out of the 3 tests conclude that a feature follows a normal distribution, it is standardized by removing the mean and scaling to unit variance [20-22]. Otherwise, features are normalized using the minimum-maximum normalization technique so that all features have values between “0” and “1.” This eliminates any feature bias, where features with high values are given more importance than features with low values [23].

Once the records are cleaned, transformed, and normalized, they are then passed into the data balancer. At this point, the results differ depending on the behavioral outcome being predicted. The synthetic minority oversampling technique for nominal and continuous (SMOTE-NC) is used to artificially balance the instances where the number of patients having a behavioral outcome of “1” is the minority, which is most often the case as cancer survivor datasets are often imbalanced. The SMOTE-NC technique oversamples the minority class in unbalanced datasets by creating synthetic examples instead of oversampling using replacement. The algorithm involves computing the median of the SD of continuous variables for the minority class and using the median to penalize nominal features that differ between the considered feature vector and its potential nearest neighbors, conducting nearest neighbors computation, and populating the synthetic class [24]. The SMOTE-NC technique is also used to artificially oversample the minority gender so the final datasets can have equal instances of “0” and “1” for the behavioral outcome. We specifically chose the SMOTE-NC technique over the regular synthetic minority oversampling technique because our dataset had a mixture of nominal and continuous features. synthetic minority oversampling technique can only handle datasets with continuous features. The data were then split into 69.6% (71/102) training and 30.4% (31/102) testing data.

Once the survivors of cancer’ clinical records passed through all the steps of data preprocessing, they were passed into our hybrid deep learning–based feature selection that was a 2-stage feature selection algorithm, that is, a multimetric, majority-voting filter and a DDN, to dynamically and automatically select the best set of features for each behavioral outcome. Specifically, the first stage was a novel filter method that uses 4 metrics to select the most relevant features for a behavioral outcome and removes any redundant features. The second stage was a DDN that replaces a wrapper method, where it further selects features from the ones selected by the multimetric, majority-voting filter to maximize prediction performance in ML classifiers. Note that our clinical domain experts used their clinical expertise to recommend certain features that should be kept in all the final feature lists due to their clinical importance (ie, gender, current age, and age at diagnosis in our case), if they were not already selected to be in the final feature list by our feature selection approach. Finally, the training data with the final feature list selected from the feature selector with the clinical domain expertise were passed into 3 ML classifiers, including logistic regression, naive Bayes, and k-nearest neighbors, to calculate the precision, recall, and *F*_1_-score for the performance evaluation on the testing data.

### 2-Stage Feature Selection Algorithm

**Figure 2:**
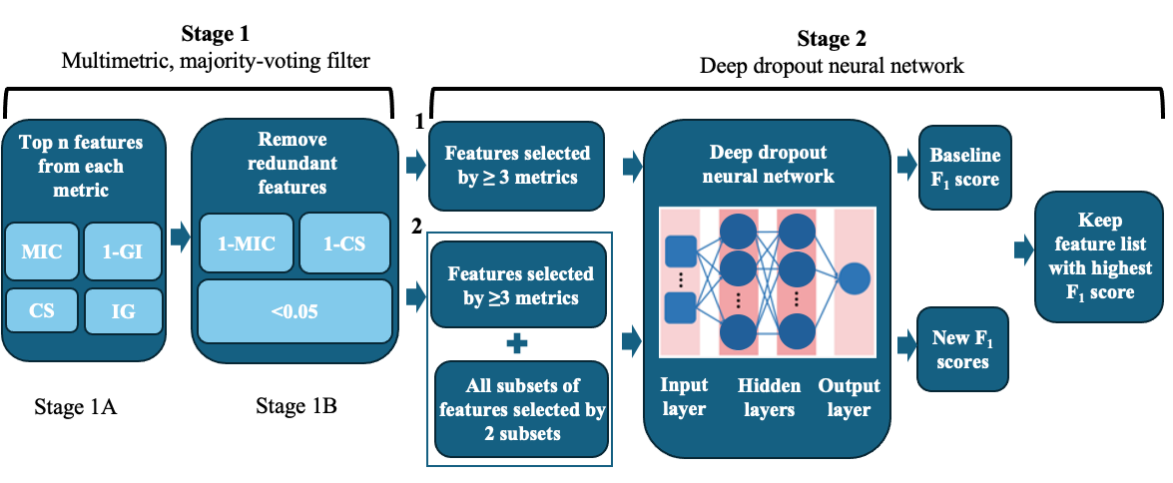
Two-stage feature selection algorithm.

Our proposed 2-stage feature selection algorithm consisted of 2 sequential stages, including a multimetric, majority-voting filter, and a DDN (Figure 2).

#### Stage 1: A Multimetric, Majority-Voting Filter

##### Overview

Our hybrid deep learning–based feature selection methodology specifically addressed the limitations of existing feature selection methods. In the first stage, it removed redundant features, which some existing filter methods do not consider. Specifically, our 3 majority-voting (MV) filter had 2 processing steps in stage 1.

In stage 1A, we used 4 different metrics to select the features that are the most relevant to predict a behavioral outcome. Those metrics include maximal information coefficient (MIC), Gini index (GI), IG, and correlation score (CS) that we calculated between each candidate feature in our preprocessed dataset and the corresponding behavioral outcome of interest. The MIC is a measure of the strength of the linear or nonlinear association between 2 variables *X* and *Y*, where *X Є R* is the input feature and *Y Є R* is the corresponding behavioral outcome.

The GI represents the amount of probability of a specific feature that is classified incorrectly when selected randomly. Unlike the other 3 metrics, a higher GI score represents lower associations with the behavioral outcome of interest. To make the scale of the correlation strength between *X* and *Y* consistent among all the metrics, the metric that we used was 1–GI instead. That is, for all the 4 metrics, a higher value indicated a higher association with the behavioral outcome of interest.

The IG is a measure of the expected reduction in entropy caused by partitioning the samples according to a specific attribute *X*.

The CS between *X* and *Y* is calculated using the Pearson correlation coefficient, point-biserial correlation, and the φ coefficient, based upon the data type of *X* and *Y* [25]. When both *X* and *Y* are the continuous variables, the Pearson correlation coefficient should be used. When comparing 1 continuous and 1 binary variable, the point-biserial correlation is used [26]. Finally, when comparing 2 binary variables, the φ is used. All these measures are values between −1 and 1, with −1 being a perfect negative correlation and 1 being a perfect positive correlation, while 0 represents no correlation. We take the absolute value of each measure so that the CS is always between 0 and 1.

After we calculated the values of all 4 abovementioned metrics between each candidate feature and the behavioral outcome of interest, we ranked the top *N* features (ie, the number of features expected by the domain experts) for each of the metrics in descending order and stored them in a master list, without repetition. From this master list, we constructed 3 feature lists. The first list contained the features selected by at least 3 metrics, as they are highly likely relevant to predict the behavioral outcome and are then included in the final feature list. The second one contained the features selected by exactly 2 metrics, as they might have been relevant to predict the behavioral outcome and were then needed for further analysis in stage 1B. The third one combined all the features from the previous 2 lists so that we could evaluate the redundancy between any 2 features from this list.

In stage 1B, we removed any redundant features from the third combined list generated from stage 1A. We used the *MIC* and the *CS* and then calculated these 2 values for all the feature-to-feature combinations in the combined feature list output from stage 1A. We subtracted the *MIC* and the *CS* values from 1 and then used the *1–MIC* and *1–CS* values to determine if any feature was redundant by other features. The threshold we set was 0.05, based upon our preliminary experimental analysis, so that any combination of 2 features that resulted in both scores being <0.05 was determined to be redundant. Once it was determined that 2 features were redundant, we looked at the number of metrics that selected the features. If one of the features was selected by fewer metrics, that feature was removed from the third combined list. If both features were selected by the same number of metrics and they were redundant, we then looked at the average rank of each feature across the 4 ranked lists by MIC, GI, IG, and CS. The feature with the lower rank was removed from the third combined list. The pseudocode algorithm is detailed for the multimetric, majority-voting filter in Multimedia Appendix 1.

For illustration, we used our dataset as an example to explain our multimetric, majority-voting filter.

##### Stage 1A: Select the Top N Features Per Metric

###### Overview

Suppose we want to select the best features for predicting the behavioral outcome, thought problems. This is our *B_Outcome*. *F* is the set of all input candidate features *F_i_* in the preprocessed clinical records. We then calculate the MIC, 1–GI, IG, and CS scores for all the candidate features in the preprocessed clinical records and our *B_Outcome*, thought problems. We store these results in 4 sets, *MIC,* *1*–*GI*, *IG,* and *CS*. In this example, our domain experts expected 15 nonredundant input candidate features to be selected; thus, *N* was set to 15.

###### Step 1

We first sorted the input features (ie, *F****_i_***s) according to their MIC, 1–GI, CS, and IG scores. Since *N* was 15, we then took the top 15 features with the highest values from the MIC set and placed them into a separate set, that is, *F_MIC_*_._ We repeated this with (1–GI), IG, and CS scores and placed the top 15 features into the corresponding sets, that is, *F_1-GI_*, *F_CS_*, and *F_IG_*. At this point, we had the following features in these sets: *F_MIC_***,** *F_1-GI_*, *F_CS_*, and *F_IG_*. As there were 15 features in each set, we had 60 features across all the 4 sets (Textbox 2).

**Total input features sorted by maximal information coefficient (MIC), 1- Gini index (GI), correlation score (CS), and information gain (IG) scores in descending order.**

**F_MIC_**

Physical fatigue>overall fatigue>cognitive fatigue>family communication>family concern>IV high-dose methotrexate (MTX)>sleep fatigue>physical activity>family conflict>parental control>family mutuality>age at cancer diagnosis>intrathecal MTX dose>noncranial radiation>cranial radiation therapy

**F_1–GI_**

Years of education>intrathecal chemotherapy>leukemia risk group>intrathecal MTX dose>living space>physical activity>cognitive fatigue>family communication>physical fatigue>family mutuality>IV high-dose MTX>sleep fatigue>family conflict>age at cancer diagnosis>age at evaluation

**F_CS_**

Physical fatigue>overall fatigue>cognitive fatigue>family communication>IV high-dose MTX>family concern>sleep fatigue>family conflict>parental control>physical activity>cranial radiation therapy>noncranial radiation>intrathecal MTX dose>years of education>family mutuality

**F_IG_**

Impulsivity (on continuous performance test [CPT; Conner continuous performance test to measure a person’s performance in attention, particularly in areas of inattentiveness, impulsivity, variation in response speed, sustained attention, and information processing efficiency] attention test)>inattentiveness (on CPT Attention test)>information processing efficiency (on CPT attention test)>hematopoietic stem cell transplant>response speed variability (on CPT Attention Test)>surgery>sustained attention (on CPT attention test)>physical fatigue>overall fatigue>neurological complications>leukemia risk group >living space>inattentiveness (on CPT attention test)>inflammatory interleukin-7

###### Step 2

We then created a new set *F_UNION_*, the union of sets *F_MIC_*, *F_1–GI_*, *F_CS_*, and *F_IG_* in step 1, allowing duplicate values. This set *F_UNION_* represents all the features that have the top 15 MIC, 1–GI, IG, and CS scores. At this point, the set *F_UNION_* contained 60 total features.

###### Step 3

From the set *F_UNION_,* we created the subset *3Metrics+* from the features that were stored in at least 3 of these 4 sets, *F_MIC_*, *F_1−GI_*, *F_CS_,* and *F_IG_*. These features were then selected as 1 of the top 15 by at least 3 out of the 4 metrics, so these are likely to be highly relevant to predict our *B_Outcome*, thought problems, and were included in the final feature list. By applying this concept, the subset *3Metrics+* contained 10 features.

###### Step 4

From the set *F_UNION_,* we also created a subset *2Metrics* from features that were stored in exactly 2 out of these 4 sets, *F_MIC_*, *F_1–GI_*, *F_CS_,* and *F_IG_.* These features were selected as the top 15 by 2 out of the 4 metrics only. Thus, they may be relevant to predict the *B_Outcome*, thought problems, but needed to be further analyzed in stage 2 to determine if they should be kept in the final feature list. By applying this concept, the subset *2Metrics* contained 8 features only.

###### Step 5

We created another set *3+2Metrics*, that is, the union of the sets *3Metrics+* and *2Metrics*, without the duplicate values. At this point, the set *3+2Metrics* contained 18 features, including 10 in the *3Metrics+* set and 8 in the *2Metrics* set (Textbox 3).

**Features in the 3Metrics+ and 2Metrics sets.**

**3Metrics+**

- Physical fatigue
- Overall fatigue
- Cognitive fatigue
- Family communication
- Sleep fatigue
- Family conflict
- Family mutuality
- Physical activity
- IV high-dose methotrexate (MTX)
- Intrathecal MTX dose

**2Metrics**

- Leukemia risk group
- Living space
- Family concern
- Cranial radiation therapy
- Years of education
- Family control
- Age at cancer diagnosis
- Noncranial radiation

###### Step 6

We also created a 1D matrix, *Rank*, which stored the average rank position of each feature in *3+2Metrics* from the sets *F_MIC_, F_1–GI_, F_CS_*, and *F_IG_.* For instance, if we consider the feature “physical fatigue,” as its position was 1, 9, 1, and 9 in the sets *F_MIC_, F_1-GI_, F_CS_*, and *F_IG_,* respectively, its average position value in *Rank* was equal to 5.

###### Step 7

Finally, we evaluated whether there were too many or too few features at this stage. We first evaluated the number of features in *3Metrics+*. As *3Metrics+* had 10 features, which was less than N, there was no need to remove any extra features. We then evaluated the number of features in *3+2Metrics*. As there were 18 features in *3+2Metrics*, which was greater than **N**, there was no need to go back to step 1 to find at least 15 features. We now had 3 sets as the outputs: *3Metrics+* with 10 features that were selected by at least 3 metrics; *2Metrics* with 8 features that were selected by exactly 2 metrics; and *3+2Metrics*, with 18 features that included the features from both *3Metrics+* and *2Metrics*.

##### Stage 1B: Remove Redundant Input Features

At this step, we wanted to remove any redundant features from the features that we selected in stage 1A.

###### Step 1

We computed 1–MIC(f*_i_*, f*_j_*) values and 1–CS(f*_i_*, f*_j_*) values by the developed *compute_MIC* and *compute_CS* functions between any pair of 2 features f*_1_* and f*_2_* in 3+2Metrics. We stored the 1–MIC(f*_i_*, f*_j_*) values and 1–CS(f*_i_*, f*_j_*) values in the sets *MIC_Feature_Score* and *CS_Feature_Score*, respectively.

###### Step 2

We iterated each value in *MIC_Feature_Score* and *CS_Feature_Score* between any pair of 2 features f*_1_* and f*_2_* in *3+2Metrics* and checked if any values were <0.05. We then checked if there was any feature pair that had values <0.05 in both *MIC_Feature_Score* and *CS_Feature_Score*. Suppose we found that the values in *MIC_Feature_Score* and *CS_Feature_Score* that corresponded to the feature pair, “cranial radiation therapy” and “noncranial radiation,” were indeed both <0.05, then we select those 2 features as the feature pair that we need to further analyze, as they were categorized as the redundant features at this step. Suppose that “cranial radiation therapy” and “noncranial radiation” were both in the set *2Metrics*, meaning that they were both selected by 2 metrics, then according to the algorithm, they were selected by an equal number of metrics and we must compare their rankings in *Rank* to decide which one must be removed. Suppose that “noncranial radiation” had a lower rank, or a higher score, compared to “cranial radiation therapy,” then we remove “noncranial radiation” from the set *3+2Metrics*.

###### Step 3

After we removed the redundant features from the set *3+2Metrics*, we then split the set *3+2Metrics* into 2 new sets: *F_3M+_,* such that its nonredundant features were selected by at least 3 metrics in the set *F_UNION_,* and *F_2M_,_,_* such that its nonredundant features were selected by exactly 2 metrics in the set *F_UNION._* 

###### Step 4

We now had 2 sets: *F_3M+_* and *F_2M_*. The set *F_3M+_* had 10 features and the set *F_2M_* had 7 features after we removed “noncranial radiation” (Textbox 4).

**Nonredundant features in the set *F_3M+_* and set *F_2M_*.**

**F_3M+_**

- Physical fatigue
- Overall fatigue
- Cognitive fatigue
- Family communication
- Sleep fatigue
- Family conflict
- Family mutuality
- Physical activity
- IV high-dose methotrexate (MTX)
- Intrathecal MTX dose

**F_2M_**

- Leukemia risk group
- Living space
- Family concern
- Cranial radiation therapy
- Years of education
- Parental control
- Age at cancer diagnosis

At this step, we checked if the sum of features from *F_3M+_* and *F_2M_* was<25. After removing redundant features, we still had 17 features, which was greater than N=15; thus, we do not need to go back to step 1 in stage 1A to find at least 15 features. We can then proceed to stage 2.

#### Stage 2: A DDN

##### Overview

In the second stage, the deep neural network had a dropout parameter, where neurons are randomly ignored during construction of the neural network, to avoid model overfitting, which is a problem that the existing wrapper methods have. Thus, our methodology is better suited for finding the best features from the high-dimension, low-sample size dataset. More specifically, after the features were processed by our multimetric majority-voting filter, we passed all the nonredundant features to the deep dropout neural (DDN) network that was designed to determine whether adding any of those features selected by the only 2 metrics to the list of the features selected by at least 3 metrics resulted in a higher *F*_1_-score. Note that this step was not conducted if the number of the nonredundant features, that is, those features that were already selected by at least 3 metrics in stage 1, had met the domain experts’ expectation. Our designed DDN network was a 2-hidden– and 1-output–layer architecture. Due to the limited number of patients’ medical records with many input features, our DDN network was likely to quickly overfit a training dataset. To address this issue, we used the grid search algorithm with the *K*-fold cross-validation (CV) to find the best dropout rate for our network. We also dynamically set the network’s hidden layer size using the formula 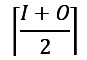, where *I* is the number of selected input subset features and *O* is the number of labels per behavioral outcome [27]. For the remaining network’s initialization parameters, default values were used [28]. The goal was to perform the hyperparameter tuning using the grid search algorithm with the *K*-fold CV to obtain the optimal parameters’ values, including the dropout rate, all the network’s parameters, and the size of each hidden layer [29].

Specifically, the subset of features selected by ≥3 metrics in stage 1 was used in building the initial network architecture to produce the baseline *F*_1_-score. This baseline *F*_1_-score tells us how well the network predicts that a cancer survivor will develop the behavioral outcome of interest, using only the features selected by at least 3 metrics. Afterward, we wanted to see whether adding any subset of features selected by 2 metrics would improve the baseline *F*_1_-score. To achieve this, we tried different combinations among the features selected by 2 metrics; added them on top of the features selected by at least 3 metrics; used all those features to build, train, and optimize our network using the grid search algorithm with the *K*-fold CV to obtain the optimal parameters’ values; and then recorded each new *F*_1_-score. This allowed us to compare *F*_1_-scores between the baseline and the baseline plus additional subsets of features. If any of the new *F*_1_-scores were higher than the baseline, then our final feature list was the one that produced the highest *F*_1_-score. If none of the new *F*_1_-scores were higher than the baseline, then our final feature list was simply the baseline features, that is, the features selected by at least 3 metrics. A step-by-step pseudocode algorithm for our DDN network is detailed in Multimedia Appendix 2).

Let us use our dataset as an example to explain our DDN network. At this stage, we wanted to determine whether any features selected by 2 metrics should be kept in the final feature list on top of the features selected by at least 3 metrics. Our input included the following:

1. *F_3M+_* and *F_2M_*, which were our outputs from stage 1B.
2. *Drop_Out_Rate*, a set of fine-tuning dropout rates for building a DDN network.
3. *D_Train*, which was the training dataset that only included features in *F_3M+_*
4. Z, the set that included all possible subsets from *F_2M_*, excluding the null set, where the size of subsets was less than or equal to N minus the size of *F_3M+_* so that the total number of features does not exceed N. In our example, the set Z only included all the possible subsets of size ≤5 because we already had 10 features in non_redundant_three_more and N minus 10 was 5. Given that there were 7 features in non_redundant_two, there were 128 possible subsets. However, because we only needed the subsets with size ≤5 and we also excluded the null set, we ended up with a total of 119 different subsets in the set Z.
5. M, a set of lists that add all the possible subsets in the set Z to the set *F_3M+_*; thus, there were 119 different lists.
6. *E_Train*, which is the set of training datasets that includes features in each list in M.
7. *K*, the number of training partitions on *D_Train* and *E_Train* for performing CV.

##### Step 1

We wanted to find the best dropout rate for the neural network, using the *grid-search* technique, *F*_1_-score, and *K*-fold CV, on *D_Train*, *F_3M+,_ B_Outcome*, and *Drop_Out_Rate* of a DDN network. *K* was set to 5. We thus first constructed a neural network using the *create_DDN* function to perform the *grid-search* technique. The neural network was initialized to have a learning rate of 0.001, 500 epochs, used the “Adam” optimizer, used the “Binary Cross Entropy” loss function, had 2 hidden layers with 
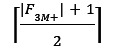
 number of neurons and the “Relu” activation function, and 1 output layer with 1 neuron and the activation function “Sigmoid.” Suppose using the *grid-search* technique with the *D_Train* training dataset, the *F_3M_****_+_*** feature set***_,_*** the *B_Outcome* thought problems, the set of fine-tuning dropout rates *Drop_Out_Rate*, and using 5-fold CV, we found that the best dropout rate was 0.1 (*bestDropOutRate* was set to 0.1).

##### Step 2

We constructed a deep neural network with the initialized attributes in the *create_DDN* function, *bestDropOutRate*, *D_Train, F_3M+_,* and *B_Outcome*, and then performed 5-fold CV to obtain the baseline *F*_1_-score, F1_Baseline_.

##### Step 3

We then iterated through each feature set (ie, *F_3M+_*+*Z_r_*) in *M* and constructed a deep neural network with the same initialized attributes in the *create_DDN* function, *bestDropOutRate*, *E_Train, F_3M+_*+*Z_r_*, and *B_Outcome,* and then performed 5-fold CV to obtain the *F*_1_-score, F1, for each training dataset in *E_Train*. The hidden layer size of each neural network was calculated using the number of features in M+1, divided by 2. If any *F*_1_-score was greater than F1_Baseline_, the final feature list (ie, *Final_Features*) was set to the feature set (ie, *F_3M+_*+*Z_r_*) in M in which the *F*_1_-score was obtained.

##### Step 4

We had the feature list with the best *F*_1_-score (ie, *Final_Features*), which was passed into 3 ML classifiers: logistic regression, naive Bayes, and k-nearest neighbors.

#### Pilot Experimental Study

In our experimental study, we used a 2018 to 2020 dataset that contained 102 ALL survivors’ clinical records collected from a public hospital in Hong Kong. The survivors were aged between 15 and 39 years, had completed treatment, and were >5 years postcancer diagnosis at the time of recruitment. In each patient record, there were >50 features, including demographic factors (eg, age, gender, and education level), cancer treatments received (eg, radiation, chemoradiotherapy, and surgery), inflammatory biomarkers (eg, interleukin-7, monocyte chemoattractant protein-1, and tumor necrosis factor alpha-α), physical health conditions (eg, BMI, sleep fatigue, and cognitive fatigue), family life and socioeconomic descriptors (eg, family conflict, family communication and living space), attention-related outcomes (eg, measures of inattentiveness, impulsivity, and sustained attention), and lifestyle habits (eg, drinking, smoking, and physical activity). The features were obtained from a behavioral assessment that included the traditional Chinese version of the Achenbach System of Empirically Based Assessment youth self-report checklist. It consisted of syndrome scales measuring attention problems, thought problems, internalizing problems (eg, somatic complaints, anxiety and depressive symptoms, and withdrawn behavior), externalizing problems (eg, aggressive behavior, intrusive behavior, and rule-breaking behavior), and sluggish cognitive tempo. The Achenbach System of Empirically Based Assessment measures were previously validated and used in the local young adult cancer population [9,30]. The inclusion of these features specifically in patient records was based on existing evidence in the literature and data from the local study cohort. The features predicting behavioral outcomes included clinical factors (eg, leukemia risk group, age at cancer diagnosis, and neurological complications), treatment factors (eg, cranial radiation therapy, intrathecal methotrexate dose, intravenous high-dose methotrexate, and hematopoietic stem cell transplant), socioenvironmental factors (eg, living space and family functioning), and lifestyle factors (eg, physical activity and sleep fatigue) [9,30-34].

After preprocessing the data and using our 2-stage feature selection algorithm, we selected 15 input features, expected by our medical investigators, to train and test our 3 ML classifiers, that is, logistic regression, naive Bayes, and k-nearest neighbors, to predict 6 behavioral outcomes (ie, anxiety and depression, thought problems, attention problems, internalizing problems, externalizing problems, and sluggish cognitive tempo) that our medical investigators would like to focus on. Due to their clinical importance recommended by our medical investigators, we also added 3 more clinically relevant features (ie, gender, current age, and age at diagnosis) to the final feature list if those features had not been already selected by our 2-stage feature selection approach.

#### Ethical Considerations

Approval of this study was obtained from the Joint Chinese University of Hong Kong – New Territories East Cluster Clinical Research Ethics Committee (2017.701). Written informed consent was obtained from all participants.

## Results

### Overview

The experimental results included the *F*_1_-score, precision, and recall on the testing data (Table 1). Note that for each feature selection method category, those scores are the average values of prediction performance among all the 3 ML classifiers for every behavioral outcome.

**Table 1:** Average F1-scores.

Behavioral outcome		Filter	Wrapper	Embedded	Hybrid	Our method	Percentage change (our method vs highest baseline)
**Anxiety and depression**							
	*F*_1_-score	0.624	0.437	0.585	0.449	*0.738* ^a^	+18.27
	Precision score	0.562	0.407	0.563	0.424	*0.708*	+25.75
	Recall score	0.813	0.519	0.630	0.580	0.778	–4.31
**Thought problems**							
	*F*_1_-score	0.490	0.438	0.477	0.394	*0.511*	+4.29
	Precision score	0.522	0.385	0.590	0.496	0.448	–24.07
	Recall score	0.537	0.556	0.463	0.383	*0.611*	+9.89
**Attention problems**							
	*F*_1_-score	0.348	0.417	0.440	0.350	*0.568*	+29.10
	Precision score	0.290	0.360	0.350	0.329	*0.515*	+43.10
	Recall score	0.463	0.519	0.630	0.424	*0.667*	+5.87
**Internalizing problems**							
	*F*_1_-score	0.533	0.706	0.619	0.637	0.700	–0.85
	Precision score	0.583	0.665	0.665	0.668	0.618	–7.49
	Recall score	0.587	0.762	0.651	0.651	*0.857*	+12.47
**Externalizing problems**							
	*F*_1_-score	0.219	0.459	0.267	0.265	0.278	–39.43
	Precision score	0.230	0.417	0.278	0.297	*0.444*	+6.47
	Recall score	0.222	0.556	0.259	0.259	0.222	–60.07
**Sluggish cognitive tempo**							
	*F*_1_-score	0.560	0.463	0.582	0.489	*0.639*	+9.79
	Precision score	0.542	0.409	0.577	0.494	0.570	–1.21
	Recall score	0.654	0.568	0.617	0.568	*0.741*	+13.30

^a^Italicized values indicate that our score was higher than the other 4 methods.

Our 2-stage feature selection approach outperformed or leveled the existing feature selection methods to support the prediction of 5 out of 6 behavioral outcomes (ie, anxiety and depression, thought problems, attention problems, internalizing problems, and sluggish cognitive tempo) in terms of the average *F*_1_-scores (Table 1). Although the wrapper method outperformed our feature selection approach to support the prediction of externalizing problems, our approach’s performance was more stable, as the *F*_1_-score variance was smaller. Thus, our feature selection approach still outperforms the other 3 existing feature selection methods.

In addition, our feature selection approach outperformed or leveled the existing feature selection methods to support the prediction of 5 out of 6 behavioral outcomes (ie, anxiety and depression, attention problems, internalizing problems, externalizing problems, and sluggish cognitive tempo) in terms of precision scores (Table 1). Although the embedded method outperformed our feature selection approach to support the prediction of thought problems, our approach’s performance variance was much smaller, which implies our approach was more stable.

Finally, our feature selection approach outperformed the existing feature selection methods to support the prediction of 4 out of 6 behavioral outcomes (ie, thought problems, attention problems, internalizing problems, and sluggish cognitive tempo) in terms of recall scores (Table 1). Although the filter and wrapper method outperformed our feature selection approach to support the prediction of anxiety and depression and externalizing problems, our approach’s performance variance was much smaller as well.

As the *F*_1_-scores were calculated from both precision and recall scores, we can infer that our feature selection approach improves the *F*_1_-scores largely because it increases the recall scores as opposed to the precision scores (Table 1). Overall, the experimental results show promising evidence that our method improves the ML classifiers’ prediction performance to support better early detection of long-term behavioral outcomes in survivors of cancer.

### Radial Feature Charts

**Figure 3:**
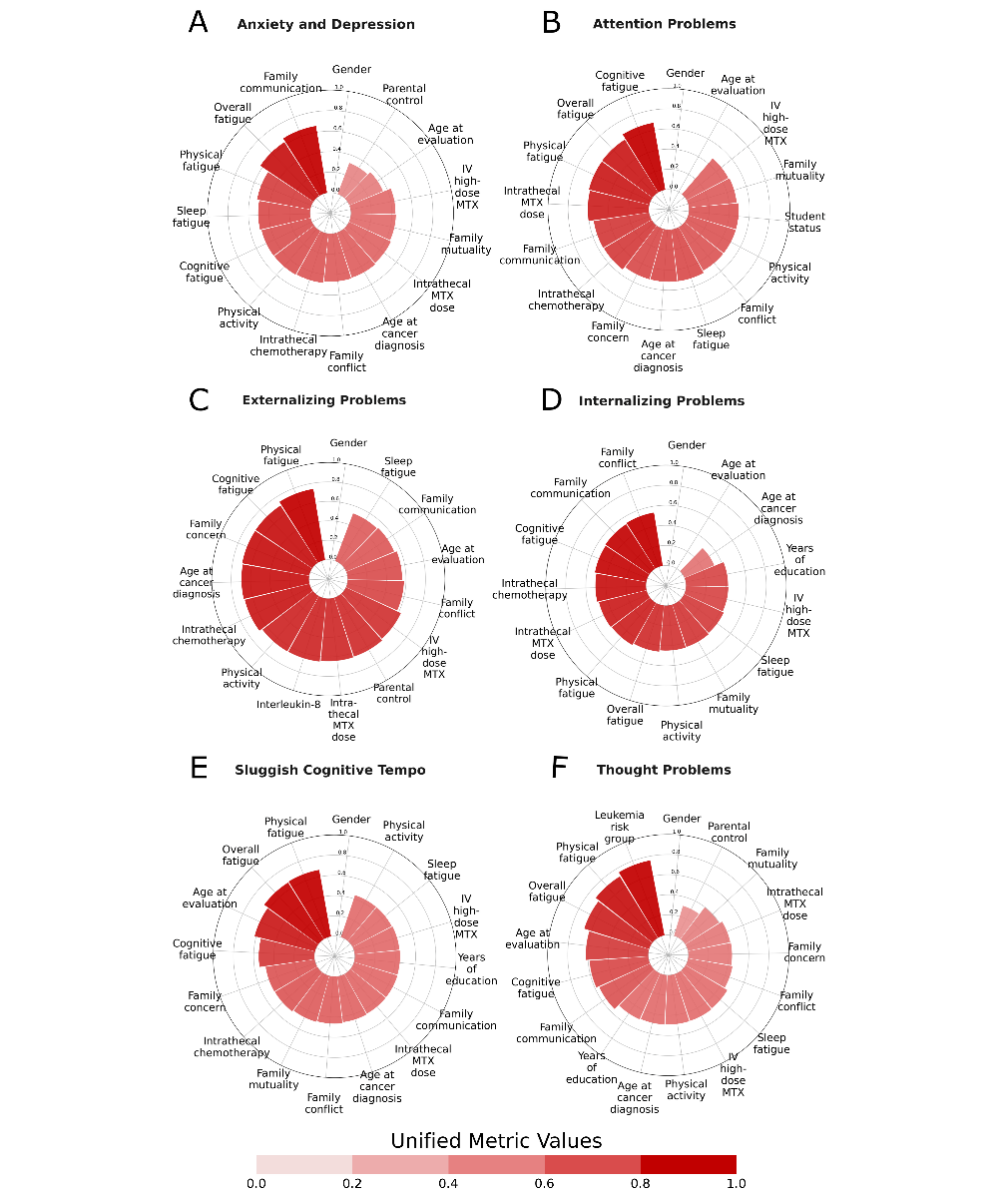
Radial feature charts.

Radial feature charts were generated for each of the 6 behavioral outcomes analyzed, including anxiety and depression, thought problems, attention problems, internalizing problems, externalizing problems, and sluggish cognitive tempo (Figure 3). Each chart includes the top 15-plus features selected by our proposed methodology. The size and the color of each red slice is measured by the unified metric value of each feature, which is calculated by averaging the scores of the metrics that select each feature during stage 1A of our proposed method.

The variables represent the documented risk factors associated with the development of behavioral problems in the literature. They include (1) sociodemographic variables (ie, age at evaluation and gender), (2) clinical variables (ie, age at cancer diagnosis, intrathecal chemotherapy, intrathecal methotrexate dose, IV high-dose methotrexate, and inflammatory interleukin-8 levels), and (3) socioenvironmental and lifestyle variables (ie, sleep, fatigue, physical activity, and family functioning). Physicians can interpret the charts by seeing which features have the darkest color and largest size, indicating higher unified metric values and thus greater associations with the behavioral problem of interest. Those features can then be further used to devise customized prevention plans and advice.

## Discussion

### Principal Findings

In this work, we sought to develop a prognostic ML framework and feature selection approach to predict the trajectory of functional outcomes in a specific population: survivors of ALL. Our hybrid deep learning–based feature selection approach outperforms or equals the existing feature selection methods assessed (ie, filter, wrapper, embedded, and hybrid) for 5 out of 6 long-term behavioral outcomes. Even in cases where our feature selection method did not outperform existing methods, our approach’s performance variance was much smaller and thus more stable. We observed that the performance of the model was significantly weaker in predicting externalizing problems than internalizing problems. This may be attributed to the complex phenotypic nature of externalizing behaviors, such as antisocial or aggressive behaviors and conduct problems. In addition, there are other factors that may predict externalizing problems that were not considered in this study. For example, our previous work showed that increased screen time during the COVID-19 pandemic was associated with inattentiveness and impulsivity in pediatric survivors of cancer in China, but screen time was not included in the data [35]. Social support and rehabilitation, which are important interventions addressing behavioral functioning and mental health in young Chinese survivors of cancer, were also not assessed in this study [36]. From the data, we infer that our feature selection approach improves *F*_1_-scores from ML classifiers compared to existing feature selection methods largely because it increases the recall scores as opposed to the precision scores. We also developed radial feature charts that can quickly and effectively help clinicians understand which predictor variables were most important in predicting long-term behavioral outcomes. Overall, the experimental results show promising evidence that our method improves ML classifiers’ prediction performance on high-dimension low-sample size data, which can support better early detection of long-term behavioral outcomes in survivors of cancer.

### Limitations

Our study was limited to a pilot study with young Chinese survivors of leukemia. As one’s neurodevelopment and social skills are often dependent on cultural norms, our findings may not be extrapolated or applicable to other populations. However, the contemporary treatment for childhood ALL is similar in most countries or regions, consisting of high-dose methotrexate, intrathecal chemotherapy, and a standard set of intravenous and oral chemotherapy drugs as the backbone. Therefore, we reasoned that our findings may still be generalizable to the existing population of individuals in the health care system of Hong Kong who have survived leukemia over the past decade. In addition, although clinical domain experts assisted with additional input for the features that were kept in ML classifiers, there remains room for human error, and domain experts’ opinions may occasionally differ from what features would optimize ML classifiers’ performance. Furthermore, as this is a cross-sectional study, it was not possible to delineate the causal relationship between the risk factors and behavioral outcomes. The model developed through this study should be validated in a larger cohort with prospective collection of outcome data to better reflect the trajectories of functional outcomes in these young survivors as they advance from young to middle adulthood. Finally, additional biases may have influenced the data, such as those related to patients who had access to hospital care and were willing to share their data with our clinical investigators.

### Comparison With Prior Work

Our findings reinforce existing evidence that adverse behavioral outcomes in survivors of cancer are a complex and multifactorial phenotype. Most preexisting research is focused on either disease- or treatment-related factors as predictors of cognitive dysfunction. However, socioenvironmental factors play an important role in the neurodevelopment of these young survivors. Our findings showed the interaction and unique contribution of the socioenvironmental factors, such as family dynamics and lifestyle factors, on anxiety, depression, and sluggish cognitive symptoms in survivors. Studies have found associations of parents’ psychological distress on the child’s cognitive and behavioral outcomes [8,37]. Environmental events can elicit a biological stress response that results in neurological reactions to that stress. This is especially relevant in the context of Hong Kong and Mainland China, where much emphasis is now placed on ameliorating the adverse health effects of the urban environment in children and adolescents. The findings provide directions for the development of multidisciplinary services and interventions. For example, social workers can pay more attention to the occupational or employment challenges of young survivors who experience fatigue symptoms from treatment and manifest adverse behavioral outcomes. The study findings can help us identify high-risk subgroups from dysfunctional families or households struggling with financial problems and conflicts. Interventions that promote self-confidence and positive peer interaction can be implemented during the early survivorship phase when young survivors transit back to their full-time school or work.

Our results also build upon existing computational methods and feature selection approaches for predicting behavioral outcomes in survivors of cancer. Traditional computational methods in the clinical and social sciences typically use regression analysis to model the relationship between ≥2 variables for prediction. However, modeling human behavioral data is challenging due to its multifactorial nature, heterogeneity, nonlinearity of data, and class imbalance [10,11]. As a result, the model can only account for a small proportion of variance, with limited utility in clinical settings. For example, we have reported that cranial radiation, chronic health conditions, and poor physical activity are associated with worse cognitive and behavioral outcomes in Chinese survivors of childhood leukemia [9]. However, these factors only accounted for 22.9% to 35.8% of the variance in the traditional regression models. Identifying an effective computational method that minimizes algorithmic bias, such as the 2-stage feature selection algorithm within the clinical domain–guided framework outlined in this study, can maximize the use of clinical and behavioral data for predictive purposes. Such prognostic models will aid in informing strategies aimed at changing behavior and designing social and clinical interventions.

### Conclusions

Future studies can validate our prediction model in other Chinese populations of survivors of cancer sharing similar cultural norms in mainland China and Taiwan, as well as validate the model in larger samples with a longitudinal prospective cohort study design. In addition, studies can further investigate the real-world feasibility of incorporating such algorithms into health care systems as risk stratification tools to assist clinicians and psychologists in identifying patients at risk of adverse behavioral outcomes. Incorporation of diverse populations, larger sample sizes, and similar prediction models in future studies may provide deeper insights into the interaction among clinical, treatment, socioeconomic, and lifestyle factors and their impact on functional outcomes, ultimately enabling the incorporation of such multifactorial insights to improve strategies for the personalized care of patients with cancer.

Given that we are working with such small cancer survivor datasets, even a slight improvement in prediction performance from ML classifiers can make a substantial difference in helping survivors of cancer. Our data-driven, clinical domain–guided approach can potentially address the problem of “high dimension low sample size.” The pilot analysis shows that this approach has allowed us to identify a set of interacting clinical and socioenvironmental characteristics that predicted behavioral outcomes in survivors.

In late 2019, the American Cancer Society had a special call for attention to financial, social, and emotional concerns that uniquely affect young survivors of cancer [38]. Currently, in Hong Kong, there are no centralized cancer programs for adolescent and young adult patients. From a clinical perspective, identifying the unique factors associated with interindividual differences in functional outcomes will help clinicians to identify individualized modifiable risk factors. This will contribute to the development of a personalized, patient-centered cancer care program for local patients with cancer. From a research perspective, this project serves as a pilot study to apply ML-based prognostic technology, guided by clinical knowledge, on a combination of objective data (ie, clinical and demographics variables) and subjective data (ie, behavioral and patient-reported variables). The framework and algorithms developed through this analysis can be applied to address clinically relevant research questions in patients with other chronic diseases. The aim of this application is in line with the recent call by the government of the Hong Kong Special Administrative Region to harness data-driven analytics to formulate health care policies [39].

## Appendix

Multimedia Appendix 1 Step-by-step pseudocode algorithm for the multimetric, majority-voting filter.

Multimedia Appendix 2 Step-by-step pseudocode algorithm for the DDN network.

## Abbreviations

ALL: acute lymphocytic leukemia
CFS: correlation-based feature selection
CS: correlation score
CV: cross-validation
DDN: deep dropout neural network
GI: Gini index
IG: information gain
Lasso: least absolute shrinkage and selection operator
MIC: maximal information coefficient
ML: machine learning
MRMR: maximum relevance minimum redundancy
SBS: sequential backwards selection
SFS: sequential forward selection
SMOTE-NC: synthetic minority oversampling technique for nominal and continuous
SS: stepwise selection
