# Supplementary material for: A Hybrid Deep Learning–Based Feature Selection Approach for Supporting Early Detection of Long-Term Behavioral Outcomes in Survivors of Cancer: Cross-Sectional Study
Source: JMIR Bioinform Biotechnol. 2025 Mar 13;6:e65001. doi: 10.2196/65001 (PMC11950700; doi:10.2196/65001)
Supplement: Multimedia Appendix 1 [file bioinform_v6i1e65001_app1.docx]

**Multimedia Appendix 1.** Step-by-step pseudocode algorithm for the multimetric, majority-voting filter.

| *Input:*  *F*: [*F_1_*, *F_2_*, ..., *F_k_*], where *F* is a set of all input candidate features *F_i_* of the pre-processed clinical records, for *1* ≤ *i* ≤ *k* and *k* ∈ *Z^++^*  *B_Outcome*: Behavioral Outcome  *D_Train*: Training Data Set on *F*  *MIC*: [*MIC_1_*, *MIC_2_*, ..., *MIC_k_*], where *MIC* is a set of the corresponding maximal information coefficient values *MIC_i_* between *F_i_* and *B_Outcome* computed on *D_Train*, for *1* ≤ *i* ≤ *k*, *MIC_i_* ∈ *MIC*, and *k* ∈ *Z^++^*  *1-GI*: [*1-GI_1_*, *1-GI_2_*, ..., *1-GI_k_*], where *1-GI* is a set of the corresponding *1-Gini Index_i_ (GI_i_)* values between *F_i_* and *B_Outcome* computed on *D_Train,* for *1* ≤ *i* ≤ *k*, *1-GI_i_* ∈ *1-GI*, and *k* ∈ *Z^++^*  *CS*: [*CS_1_*, *CS_2_*, ..., *CS_k_*], where *CS* is a set of the corresponding correlation score values *CS_i_*, i.e., Pearson Correlation Coefficient (PCC), Point Bi-Serial Correlation (PBC), or φ-Coefficient (φ), between *F_i_* and *B_Outcome* computed on *D_Train* based upon the data type of *F_i_* and *B_Outcome* for *1* ≤ *i* ≤ *k*, *CS_i_* ∈ *CS*, and *k* ∈ *Z^++^*  *IG*: [*IG_1_*, *IG_2_*, ..., *IG_k_*], where *IG* is a set of the corresponding Information Gain *IG_i_* values between *F_i_* and *B_Outcome* computed on *D_Train*, for *1* ≤ *i* ≤ *k*, *IG_i_* ∈ *IG*, and *k* ∈ *Z^++^*  *N*: The Number of Non-redundant Input Candidate Features *F_i_* Expected by Domain Experts, where *F_i_* ∈ *F*, *N* ≤ *k*, and *k* ∈ *Z^++^*  Output:  *F_3M_****_+_***: [*T_1_*, *T_2_*, …, *T_p_*], where *F_3M_****_+_*** is a set of all non-redundant input candidate features *T_j_* selected by at least three metrics, i.e., *MIC_j_*, *1-GI_j_*, *CS_j_*, and *IG_j_*, for *1* ≤ *j* ≤ *p*, *T_j_* ∈ *F*, *MIC_j_* ∈ *MIC, 1-GI_j_* ∈ *1-GI, CS_j_* ∈ *CS, IG_j_* ∈ *IG,* and *p* ∈ *Z^++^*  *F_2M_*: [*S_1_*, *S_2_*, …, *S_q_*], where *F_2M_* is a set of all non-redundant input candidate features *S_ℓ_* selected by exactly two metrics, i.e., *MIC_ℓ_*, *1-GI_ℓ_*, *CS_ℓ_*, and *IG_ℓ_*, for *1* ≤ *ℓ* ≤ *q*, *S_ℓ_* ∈ *F*, *MIC_ℓ_* ∈ *MIC, 1-GI_ℓ_* ∈ *1-GI, CS_ℓ_* ∈ *CS, IG_ℓ_* ∈ *IG,* and *q* ∈ *Z^++^*  Initialization:  3Metrics+ = [] # Store a set of candidate features *F_i_* selected by at least three metrics, i.e., *MIC_i_*, *1-GI_i_*, *CS_i_*, or *IG_i_*, where *1* ≤ *i* ≤ *k*, *F_i_* ∈ *F*, *MIC_i_* ∈ *MIC, 1-GI_i_* ∈ *1-GI, CS_i_* ∈ *CS, IG_i_* ∈ *IG,* and *k* ∈ *Z^++^*  2Metrics = [] # Store a set of candidate features *F_i_* selected by exactly two metrics, i.e., *MIC_i_*, *1-GI_i_*, *CS_i_*, or *IG_i_*, where *1* ≤ *i* ≤ *k*, *F_i_* ∈ *F*, *MIC_i_* ∈ *MIC, 1-GI_i_* ∈ *1-GI, CS_i_* ∈ *CS, IG_i_* ∈ *IG,* and *k* ∈ *Z^++^*  3+2Metrics = [] # Store a set of candidate features *F_i_* from both 3Metrics+ and 2Metrics  *Rank* = [] # Store a set of mean rank positions for each feature in 3+2Metrics. The smaller the position value, the higher the feature rank.  MIC_Feature_Score = [] # Store a set of 1 – MIC[*f_i_*, *f_j_*] values between any pair of two features *f_i_* and *f_j_* in 3+2Metrics computed on *D_Train*, where *i* ≠ *j*.  CS_Feature_Score = [] # Store a set of 1 – CS[*f_i_*, *f_j_*] values between any pair of two features *f_i_* and *f_j_* in 3+2Metrics computed on *D_Train*, where *i* ≠ *j*.  M = *N #* Set the initial number of available input candidate features, where M ≥ N and M ≤ k, for M, N, k ∈ *Z^++^* |
| --- |
| Processing:  *STAGE 1A –* Select the Top *N* Features Per Metric  *STEP 1*: Sort *F_i_*s in the descending order, according to their *MIC_i_*, *1*-*GI_i_*, *CS_i_*, and *IG_i_* values, by the developed *sort_features* function and then store their corresponding top M features in the sets, i.e., *F_MIC_*_,_ *F_1-GI_*, *F_CS_*, and *F_IG_*.  *F_MIC_* = *sort_features*(*F*, by = *MIC*, ascending = *False*).top(M)  *F_1-GI_* = *sort_features*(*F*, by = *1-GI*, ascending = *False*).top(M)  *F_CS_* = *sort_features*(*F*, by = *CS*, ascending = *False*).top(M)  *F_IG_* = *sort_features*(*F*, by = *IG*, ascending = *False*).top(M)  *STEP 2*: Create a set *F_UNION_* = *F_MIC_* U_A_F_1-G1_ U_A_F_CS_ U_A_F_1G_, where U_A_ is a UNION ALL operator that can combine two or more result sets with duplicate values.  *STEP 3*: Check if a feature *F_i_* ∈ *F* appears in at least three metrics in *F_UNION_* and then store it in 3Metrics+.  for *f* in *F_UNION_*:  if COUNT(*f*) ≥ 3 in *F_UNION_*:  3Metrics+.add(*f*)  *STEP 4*: Check if a feature *F_i_* ∈ *F* appears in exactly two metrics in *F_UNION_* and then store it in 2Metrics.  for *f* in *F_UNION_*:  if COUNT(*f*) == 2 in *F_UNION_*:  2Metrics.add(*f*)  *STEP 5*: Create a set 3+2Metrics = 3Metrics+ U 2Metrics, where U is a UNION operator that can combine two or more result sets without duplicate values.  *STEP 6*: Calculate the mean ranking position of each feature *F_i_* ∈ *F* in 3+2Metrics by the developed *rank* function and then store it in the 1D matrix, i.e., *Rank*.  for *f* in 3+2Metrics:  *r_MIC_* = *rank*(*f*, *F_MIC_*)  *r_1-GI_* = *rank*(*f*, *F_1-GI_*)  *r_CS_* = *rank*(*f*, *F_CS_*)  *r_IG_* = *rank*(*f*, *F_IG_*)  *r_f_* = $\lceil\frac{r_{MIC}+r_{1-GI}+r_{CS}+r_{IG}}{4}\rceil$  *Rank*[*f*] = *r_f_*  *STEP 7*: Evaluate if the algorithm has enough input candidate features *F_i_*s, expected by domain experts, for the redundancy checking.  if size(3Metrics+) > *N*:  3Metrics+ = *sort_features*(3Metrics+, by = *Rank*, ascending = *True*)  del 3Metrics+[*N*:]  3+2Metrics = 3Metrics+ U 2Metrics  Return 3Metrics+, 2Metrics, and 3+2Metrics  elseif size(3Metrics+) + size(2Metrics) < *N*:  M = M + 1  GoTo *STEP 1*  else:  Return 3Metrics+, 2Metrics, and 3+2Metrics  *STAGE 1B* – Remove Redundant Input Features  *STEP 1:* Compute 1 – MIC[*f_i_*, *f_j_*] values and 1 – CS[*f_i_*, *f_j_*] values, by the developed *compute_MIC* and *compute_CS* functions between any pair of two features *f_1_* and *f_2_* in 3+2Metrics.  for *f_1_* in 3+2Metrics:  for *f_2_* in 3+2Metrics:  if *f_1_* ≠ *f_2_*:  MIC[*f_1_*, *f_2_*] = *compute_MIC(f_1_*, *f_2_*)  CS[*f_1_*, *f_2_*] ] = *compute_CS(f_1_*, *f_2_*)  MIC_Feature_Score[*f_1_*, *f_2_*] = 1 – MIC[*f_1_*, *f_2_*]  CS_Feature_Score[*f_1_*, *f_2_*] = 1 – CS[*f_1_*, *f_2_*]  *STEP 2*: Iterate each value in MIC_Feature_Score and CS_Feature_Score between any pair of two features *f_1_* and *f_2_* in 3+2Metrics and then remove the redundant one, i.e., MIC_Feature_Score[*f_1_*, *f_2_*] < 0.05 and CS_Feature_Score[*f_1_*, *f_2_*] < 0.05, according to their counts and ranks in 3Metrics+ and 2Metrics, where 0.05 is the defined threshold.  let Temp = 3+2Metrics  for *f_1_* in 3+2Metrics:  for *f_2_* in 3+2Metrics:  if *f_1_* ≠ *f_2_* AND MIC_Feature_Score[*f_1_*, *f_2_*] < 0.05 AND CS_Feature_Score[*f_1_*, *f_2_*] < 0.05:  if *f_1_* in 3Metrics+ AND *f_2_* in 2Metrics:  Temp.remove(*f_2_*)  elseif *f_2_* in 3Metrics+ AND *f_1_* in 2Metrics:  Temp.remove(*f_1_*)  elseif *f_1_* in 3Metrics+ AND *f_2_* in 3Metrics+:  if COUNT(*f_1_*) in *F_UNION_* > COUNT(*f_2_*) in *F_UNION_*:  Temp.remove(*f_2_*)  elseif COUNT(*f_2_*) in *F_UNION_* > COUNT(*f_1_*) in *F_UNION_*:  Temp.remove(*f_1_*)  elseif *Rank*[*f_1_*] > *Rank*[*f_2_*]:  Temp.remove[*f_2_*]  else:  Temp.remove[*f_1_*]  elseif *f_1_* in 2Metrics AND *f_2_* in 2Metrics:  if *Rank*[*f_1_*] > *Rank*[*f_2_*]:  Temp.remove[*f_2_*]  else:  Temp.remove[*f_1_*]  3+2Metrics = Temp    *STEP 3*: Split 3+2Metrics into two sets, *F_3M+_* and *F_2M_*  for *f* in 3+2Metrics:  if COUNT(*f*) >= 3 in *F_UNION_*:  *F_3M+_*.add(*f*)  else:  *F_2M_*.add(*f*)  *STEP 4*: Return *F_3M+_* and *F_2M_* if the algorithm has enough non-redundant input candidate features *F_i_*s, expected by domain experts, or go back to *STEP 1* of *STAGE 1A*.  if size(*F_3M+_*) + size(*F_2M_*) < *N*:  M = M + 1  GoTo *STEP 1* of *STAGE 1A*  else:  Return *F_3M+_* and *F_2M_* |
