# Supplementary material for: A Hybrid Deep Learning–Based Feature Selection Approach for Supporting Early Detection of Long-Term Behavioral Outcomes in Survivors of Cancer: Cross-Sectional Study
Source: JMIR Bioinform Biotechnol. 2025 Mar 13;6:e65001. doi: 10.2196/65001 (PMC11950700; doi:10.2196/65001)
Supplement: Multimedia Appendix 2 [file bioinform_v6i1e65001_app2.docx]

**Multimedia Appendix 2.** Step-by-step pseudocode algorithm for the DDN network.

| *Input:*  *N*: The Number of Non-redundant Input Candidate Features *F_i_* Expected by Domain Experts, where *F_i_* ∈ *F*, *N* ≤ *k*, and *k* ∈ *Z^++^*  *F_3M+_*: [*T_1_*, *T_2_*, ..., *T_p_*], where *F_3M+_* is a set of all non-redundant input candidate features *T_j_* selected by at least three metrics, i.e., *MIC_j_*, *1-GI_j_*, *CS_j_*, and *IG_j_*, for *1* ≤ *j* ≤ *p*, *T_j_* ∈ *F*, *MIC_j_* ∈ *MIC, 1-GI_j_* ∈ *1-GI, CS_j_* ∈ *CS, IG_j_* ∈ *IG,* and *p* ∈ *Z^++^*  *F_2M_*: [*S_1_*, *S_2_*, ..., *S_q_*], where *F_2M_* is a set of all non-redundant input candidate features *S_ℓ_* selected by exactly two metrics, i.e., *MIC_ℓ_*, *1-GI_ℓ_*, *CS_ℓ_*, and *IG_ℓ_*, for *1* ≤ *ℓ* ≤ *q*, *S_ℓ_* ∈ *F*, *MIC_ℓ_* ∈ *MIC, 1-GI_ℓ_* ∈ *1-GI, CS_ℓ_* ∈ *CS, IG_ℓ_* ∈ *IG,* and *q* ∈ *Z^++^*  *B_Outcome*: Behavioral Outcome  *Drop_Out_Rate*: [0.0, 0.1, 0.2, 0.3, 0.4, 0.5, 0.6, 0.7, 0.8, 0.9] is a set of fine-tuning dropout rates for building a DDN network.  *D_Train*: Training Data Set on *F_3M+_*  *Z*: [*Z_1_*, *Z_2_*, … *Z_n_*], where *Z* is a set of *Z_r_*s, for *Z_r_* is a possible subset combination of *F_2M_*, *Z_r_* ≠ θ, and \|*Z_r_* \| ≤ *N* - \|*F_3M+_*\|, 1 ≤ *r* ≤ *n*, and *n* ∈ Z^++^  *M*: [*F_3M+_* + *Z_r_*], where *M* is a set of *F_3M+_* U *Z_r_*, for *Z_r_* is a possible subset combination of *F_2M_*, *Z_r_* ≠ θ, and \|*Z_r_* \| ≤ *N* - \|*F_3M+_*\|, 1 ≤ *r* ≤ *n*, and *n* ∈ Z^++^  *E_Train*: Training Data Sets on *M*  *K*: The Number of Training Partitions on *D_Train* and *E_Train* for Performing Cross-Validation (CV)  *Output:*  *Final_Features*: A Set of Final Features Selected by the DDN Network for Building Machine Learning Classifiers.  *Initialization:*  Learning_Rate = 0.001 # The hyperparameter to govern the pace at which the optimizer algorithm updates the weight values of a DDN network.  Epochs = 500 # The hyperparameter to define the number of times that the learning optimizer works through the entire training dataset on a DDN network  Optimizer = "Adam" # The Adam Optimizer is used for training a DDN network  Loss_Function = "Binary Cross Entropy" # The logarithmic loss to track incorrect labeling of the data class by a DDN network and penalize the network if deviations in probability occur in classifying the labels.  Number_Of_Hidden_Layers = 2 # The number of hidden layers in a DDN network  Number_Of_Output_Layer = 1 # The number of output layer in a DDN network  Hidden_Layer_Size = Inline graphic 3 # The number of neurons in each hidden layer, where \|F_3M+_\| is the number of input features in F_3M+_  Output_Layer_Size = 1 # The number of neurons in the output layer  Hidden_Layer_Activation_Function = "Relu" # The neuron activation function used in each hidden layer  Output_Layer_Activation_Function = "Sigmoid" # The neuron activation function used in the output layer |
| --- |
| *Processing:*  *STEP 1*: Find the best dropout rate, by using the *Grid-Search* technique, F1-score, and *K*-fold cross-validation, on *D_Train*, *F_3M+_*, *B_Outcome*, and *Drop_Out_Rate* of a DDN network constructed by the *create_DDN* function.  let maxF1-score = 0  let bestDropOutRate = 0  for *dor* in *Drop_Out_Rate*:  DDN = *create_DDN*(*dor*, \| *F_3M+_*\|, Number_Of_Hidden_Layers, Number_Of_Output_Layer, Hidden_Layer_Size, Output_Layer_Size, Hidden_Layer_Activation_Function, Output_Layer_Activation_Function)  F1-score[*dor*] = DNN.train_model(*D_Train*, *B_Outcome*, *K*, Learning_Rate, Epochs, Optimizer, Loss_Function)  if F1-score[*dor*] > maxF1-score:  maxF1-score = F1-score[*dor*]  bestDropOutRate = *dor*  *STEP 2*: Construct a DDN network, by the *create_DDN* function, on bestDropOutRate, *D_Train*, *F_3M+_*, and *B_Outcome*, and then perform *K*-fold cross-validation to obtain the baseline F1 score, i.e., F1_Baseline._  *STEP 3*: Iterate each feature set [*F_3M+_* + *Z_r_*] in *M* and construct a DDN network, by the *create_DDN* function, on bestDropOutRate, *E_Train*, [*F_3M+_* + *Z_r_*], and *B_Outcome*, and then perform the *K*-fold cross-validation to obtain its F1 score, i.e., F1_r_, where 1 ≤ *r* ≤ *n*  *Final_Features = F_3M+_*  F1_Max_ = F1_Baseline_  for *fs* in *M*:  Hidden_Layer_Size = $\lceil\frac{fs+1}{2}\rceil$  DDN = *create_DDN*(bestDropOutRate, \|*fs\|*, Number_Of_Hidden_Layers, Number_Of_Output_Layer, Hidden_Layer_Size, Output_Layer_Size, Hidden_Layer_Activation_Function, Output_Layer_Activation_Function)  F1*_fs_* = DNN.train_model(*E_Train*, *B_Outcome*, *K*, Learning_Rate, Epochs, Optimizer, Loss_Function)  if F1*_fs_* > F1_Max_:  F1_Max_ = F1*_fs_*  *Final_Features = fs*  *STEP 4*: Return *Final_Features* |
